# Alteration of the gut microbiota in Chinese population with chronic kidney disease

**DOI:** 10.1038/s41598-017-02989-2

**Published:** 2017-06-06

**Authors:** Shuanghong Jiang, Shan Xie, Dan Lv, Pu Wang, Hanchang He, Ting Zhang, Youlian Zhou, Qianyun Lin, Hongwei Zhou, Jianping Jiang, Jing Nie, Fanfan Hou, Ye Chen

**Affiliations:** 1 Guangdong Provincial Key Laboratory of Gastroenterology, Department of Gastroenterology, Nanfang Hospital, Southern Medical University, State Key Laboratory of Organ Failure Research, Ministry of Education, Guangzhou, China; 2 Department of Nephrology, Nanfang Hospital, Southern Medical University, State Key Laboratory of Organ Failure Research, Ministry of Education, Research Institute of Nephrology, Guangdong Province, Guangzhou, China; 3 Department of Nephrology, The first people’s Hospital of Foshan, Guangdong Province, Foshan, China; 4 School of Public Health and Tropical Disease, Southern Medical University, North1838 Guangzhou Road, Guangzhou, 510515 P.R. China

## Abstract

We evaluated differences in the compositions of faecal microbiota between 52 end stage renal disease (ESRD) patients and 60 healthy controls in southern China using quantitative real-time polymerase chain reaction (qPCR) and high-throughput sequencing (16S ribosomal RNA V4-6 region) methods. The absolute quantification of total bacteria was significantly reduced in ESRD patients (p < 0.01). In three enterotypes, *Prevotella* was enriched in the healthy group whereas *Bacteroides* were prevalent in the ESRD group (LDA score > 4.5). 11 bacterial taxa were significantly overrepresented in samples from ESRD and 22 bacterial taxa were overrepresented in samples from healthy controls. The butyrate producing bacteria, *Roseburia*, *Faecalibacterium*, *Clostridium*, *Coprococcus* and *Prevotella* were reduced in the ESRD group (LDA values > 2.0). Canonical correspondence analysis (CCA) indicated that Cystatin C (CysC), creatinine and eGFR appeared to be the most important environmental parameters to influence the overall microbial communities. In qPCR analysis, The butyrate producing species *Roseburia spp*., *Faecalibacterium prausnitzii*, *Prevotella* and *Universal bacteria*, were negatively related to CRP and CysC. Total bacteria in faeces were reduced in patients with ESRD compared to that in healthy individuals. The enterotypes change from *Prevotella* to *Bacteroides* in ESRD patients. The gut microbiota was associated with the inflammatory state and renal function of chronic kidney disease.

## Introduction

The human gut is immensely populated with microorganisms, predominantly anaerobic bacteria. This internalized ‘Microbial organ’, which are not encoded in the host genome, consists of at least 10^13^ citizens and 500–1,000 different species whose collective genomes are estimated to contain 100 times more genes than our own human genome^1, 2^. This microbial community forms a natural defense barrier and influences nutrition (vitamin K and vitamin B12 synthesis, Energy regulation)^3, 4^, physiology^5^, immune function (mucosal immune responses, inflammatory signaling through toll-like receptors, NF-κB, etc; and adaptive Immunity through CD4 T-regulatory cell populations (Tregs))^6, 7^, and metabolism (short-chain fatty acids, bile acids, choline, indole, lipids and others)^3, 4, 8^. Various clinical and animal studies suggest that our gut microbial environment plays a critical role in both maintenance of health and disease pathogenesis, such as in obesity^9^, diabetes^10, 11^, non-alcoholic fatty liver disease (NAFLD)^10^, IBD^12^, cardiovascular disease^13^, cancers^14^, and refractory Clostridium difficile infection^15^.

Chronic kidney disease (CKD) is widespread, afflicting millions of people worldwide. In China, approximately 119.5 million adults have CKD, making it an important public health problem^16, 17^. CKD patients have enteric bacterial overgrowth and harbor. A greatly increased microbial flora comprises both anaerobes (10^7^ bacteria/mL) and aerobes (10^6^ bacteria/mL) in the duodenum and jejunum, which is quantitatively comparable to those in blind loop. However the composition of these does not differ significantly between the two groups^18^. An fecal analysis revealed a disturbed composition of microbiota characterized by an overgrowth of aerobic bacteria in hemodialysis patients^19^. Recent studies have revealed profound alterations of gut microbiota in patients and animals with CKD. Vaziri *et al*. demonstrated via 16S rRNA genePhyloChip analysis that uremia profoundly alters intestinal microbial flora^20^. Moreover some studies have suggested the pathogenic role of gut microbiota in kidney disease^21^. Alterations in the composition of the microbiome and accumulation of gut derived uremic toxins (such as lipopolysaccharides, indoxyl sulphate (IS), *p-*cresyl sulphate (PCS), amines, ammonia, and trimethylamine oxide) contribute to the systemic inflammation, cardiovascular disease and numerous other CKD associated complications^13, 22, 23^. IS and PCS were associated with elevated levels of selected inflammatory markers (serum IL-6, TNF-alpha and IFN-gamma) and an antioxidant in CKD patients^24^ and predict progression of CKD^25^. Butyrate produced from microbial fermentation is important for energy metabolism and normal development of colonic epithelial cells, mainly has a protective role in relation to colonic disease, and appears to decrease the inflammatory response^26, 27^. Smith *et al*.^28^ found that short chain fatty acids (SCFAs) regulate the size and function of the colonic Treg pool, which play a major role in the pathogenesis of systemic inflammation, maintaining immunological self-tolerance, limiting the inflammatory response to foreign antigens and protecting against colitis. Butyrate regulates the differentiation of Treg cells^29^. ESRD is compounded by the depletion and dysfunction of regulatory T lymphocytes^30^. CKD impairs the barrier function and alters microbial flora of the intestine. Bacterial translocation and uremic toxicity as possible sources contributed to the chronic inflammation noted in uremia^31, 32^. The aim of this study was to evaluate and quantify differences in the composition of gut microbiota in ESRD patients in southern China.

## Materials and Methods

### Study subjects

CKD definitions and classifications in this study are in accordance with the 2002 clinical practice guideline, end stage renal disease (ESRD) was defined as the estimated glomerular filtration rate (eGFR) less than 15 mL/min/1.73 m^2^ for 3 months, irrespective of the presence or absence of kidney damage^33^. All ESRD patients were diagnosed in accordance with this guideline by professional kidney internal medicine physicians^33^. All methods, including the collection of blood and faecal samples, were performed in accordance with the relevant guidelines and regulations. All the people have signed the informed consent. The study was reviewed and approved by the Medical Ethics Committee of the Southern Medical University, Guangzhou, China. Fresh faecal samples collected in sterile containers from 52 ESRD patients and 60 healthy volunteers (controls) were used for quantitative PCR (qPCR), of these, samples from 27 ESRD patients and 26 healthy volunteers underwent Pyrosequencing. The underlying cause of 21 ESRD patients was chronic glomerulonephritis, 11 was hypertensive nephropathy, 6 was obstructive nephropathy, 3 was polycystic kidney disease, 2 was systemic lupus erythematosus, 2 was chronic pyelonephritis, 7 was unclear. Only two of the ESRD patients have received hemodialysis therapy through deep venous catheterization for once before the enrollment because of the acute hyperkalemia. The rest patients have never been treated with dialysis. All ESRD inpatients had never been treated with dialysis or without a regular dialysis. Exclusion criteria included treatment with antibiotics, probiotics/prebiotics and other laxatives in the 4 weeks preceding sample collection. We also excluded cholecystectomy, colectomy or intestinal disease and diabetes and hyperlipidemia from our data. Clinical datas of all the subjects were shown in Table 1.

**Table 1:** Clinical parameters among ESRD patients and healthy controls.

characteristics	ESRD (n = 52)	controls (n = 60)	*p* value
Age (years)	51.58 ± 18.33	52.53 ± 13.98	0.746
sex, male (female)	29 (23)	25 (35)	0.184
BMI (kg/m^2)	22.52 ± 2.74	21.64 ± 3.25	0.098
CysC (mg/L)	6.74 ± 3.84	0.91 ± 0.14	0.000**
BUN (mmol/L)	26.65 ± 10.38	5.24 ± 1.54	0.000**
Scr (μmol/L)	654.36 ± 174.86	76.57 ± 26.89	0.000**
eGFR (ml/min/1.73 m^2^)	6.86 ± 2.87	98.03 ± 27.32	0.000**
CRP (mg/L)	19.20 ± 40.64	1.93 ± 2.58	0.005**
LPS (EU/mL)	0.11 ± 0.05	0.08 ± 0.04	0.033*
glucose (mmol/L)	5.45 ± 1.46	4.78 ± 0.60	0.089
TG (mmol/L)	1.71 ± 1.31	1.61 ± 1.21	0.669
CHOL (mmol/L)	4.80 ± 1.49	4.61 ± 0.69	0.831
VLDL (mmol/L)	0.79 ± 0.51	0.61 ± 0.0.45	0.134
LDL (mmol/L)	2.63 ± 1.15	2.76 ± 0.59	0.749
HDL (mmol/L)	1.38 ± 0.41	1.54 ± 0.41	0.194
Lpa (mg/L)	0.44 ± 0.29	0.39 ± 0.21	0.066
ApoE (mg/L)	43.17 ± 20.27	39.67 ± 27.00	0.697
ApoA, B (mg/L)	1.33 ± 0.32	1.45 ± 0.43	0.146

In this experiment, patients with diabetes and hyperlipidemia were excluded. Abbrevitions: CKD, Chronic kidney disease; BMI, body mass index; Scr, serum creatinine; CysC, Cystatin C; BUN, Blood Urea Nitrogen; eGFR, estimated glomerular filtration rate. CRP, C-reactive protein; LPS, Lipopolysaccharide; TG, Triglyceride; CHOL, Cholesterol; VLDL, very low-density lipoprotein; LDL, low-density lipoprotein; HDL, high density lipoprotein; Lpa, lipoproteins a; ApoE, apolipoprotein E; ApoA, B, apolipoprotein A, B *p < 0.05, **p < 0.01. mean ± SD.

### Assessment of clinical parameters

Fasting venous blood samples were collected in the morning, and centrifuged at 3000 g/min, at 4 °C for 10 min. The recovered supernatants was separated in 200 μL tubes and immediately frozen at −80 °C. We used the enzymatic method (isotope dilution mass spectrometry, IDMS reference method) to measure the creatinine. A modified kinetic Jaffé method was used to measure blood urea nitrogen (BUN) and the CKD Epidemiology Collaboration (CKD-EPI) equation was used to measure estimated glomerular filtration rate (eGFR) values. Cystatin C (CysC) and C-reactive protein (CRP) were measured by immunoturbidimetric assays. Lipopolysaccharide (LPS) was detected with the chromogenic end-point Limulus Amebocyte Lysate (LAL) assay. Plasma cholesterol (CHOL); triglycerides (TG); and high-density lipoprotein (HDL), low-density lipoprotein (LDL), and very-low-density lipoprotein (VLDL) cholesterol levels were determined using enzymatic methods.

### Sampling and DNA extraction

Fresh stools were collected one day after enrollment and frozen at −80°C, patients who did not have a bowel movement were excluded. Two tubes were collected and filled at least 1/3. According to the TIANamp Stool DNA Kit (TIANGEN Biotech, Beijing, China) manufacturer steps to extract the faecal DNA. All DNA samples were stored at −80 °C until further processing.

### Pyrosequencing and bioinformatics analysis

Isolated fecal DNA was used as a template for amplification of the 16S rRNA V4-6 region using the universal primer V4F (5′-GTGCCAGCMGCCGCGGTAA-3′) and V6R (5′-ACAGCCATGCNCACCT-3′). 20 μl reaction mixture: 10 μl TaKaRa Premix Taq, 2 μl template DNA, 0.5 μl 10 μM barcode forward primer, 0.5 μl 10 μM reverse primer, and 7 μl double-distilled H_2_O. The PCR cycle conditions: an initial denaturation at 94 °C for 5 min, 25 cycles of 94 °C for 30 s, 59 °C for 30 s, and 72 °C for 30 s, and a final extension at 72 °C for 5 min. PCR products were sequenced using Illumina GAII (Illumina, San Diego, CA, USA) at the Beijing Genomic Institute (Shenzhen, China). Sequencing results were clustered by lllumina paired barcoded - sequencing (end) (BIPES) (PE) process for preliminary analysis, the rest of the sequence were screened by UCHIME and removed the suspected chimeric sequence. All reads were sorted into different samples according to their barcodes. Then the two stage clustering (TSC) was used for clustering to extract the OUT in order to to distinguish the high abundance and low abundance sequences. Principal coordinates analysis (PcoA) based on UniFrac distance was performed with QIIME. The linear discriminant analysis (LDA) with effect size measurements (LEfSe) were used to identify indicator bacterial groups specialized within the two groups.

### Quantitative real-time PCR (qPCR)

The bacteria selected for qPCR are wellknown bacteria in gut. *Escherichia coli* (*E. coli*) belongs to Proteobacteria. Bacteroides fragilis group belong to Bacteroidetes. Bifidobacterium belong to Actinobacteria. Enterococcus spp., Lactobacillus group and Clostridium coccoides group belong to Firmicutes. Based on the sequencing data, Roseburia spp., Faecalibacterium prausnitzii and Prevotella which are typical butyrate producing bacteria were decreased in ESRD patients, so we chose it. All qPCR primer are listed in Table 2 ^34–38^. qPCR assays were performed in a 96-well optical plate on a LightCycler® 480 Real-Time PCR System (Roche Diagnostics, Basel, Switzerland). All assays were carried out in duplicate. The reaction mixtures consisted of 10 μl TaKaRa Premix Taq, 2 μl template DNA, 0.4 μl 10 μM barcode forward primer, 0.4 μl 10 μM reverse primer, and 7.2 μl double-distilled H_2_O. The copy number of target DNA was determined by serially diluting standards (10^1^ to 10^7^ copies of plasmid DNA containing the respective amplicon for each set of primers) running on the same plate. Bacterial quantity was expressed as log_10_ bacteria per gram of stool.

**Table 2:** Primers used for qPCR in this study.

Target Bacteria	Primer	Sequence (5^′^ to 3^′^)	Annealing (°C)	Product	Reference
Universal bacteria	Univ-F	AGAGTTTGATCATGGCTCAG	55	540	34
	Univ-R	ACCGCGACTGCTGCTGGCAC			
*E. coli*	E. col-F	GTTAATACCTTTGCTCATTGA	55	340	35
	E. col-R	ACCAGGGTATCTAATCC			
Bacteroides fragilis group	Bfra-F	ATAGCCTTTCGAAAGRAAGAT	50	501	36
	Bfra-R	CCAGTATCAACTGCAATTTTA			
Enterococcus spp.	Ente-F	CCCTTATTGTTAGTTGCCATCATT	61	144	37
	Ente-R	ACTCGTTGTACTTCCCATTGT			
Lactobacillus group	Lact-f	AGCAGTAGGGAATCTTCCA	58	341	37
	Lact-R	CACCGCTACACATGGAG			
Bifidobacterium	Bifid-F	CTCCTGGAAACGGGTGG	55	549–563	36
	Bifid-R	GGTGTTCTTCCCGATATCTACA			
Clostridium coccoides group	Ccoc-F	AAATGACGGTACCTGACTAA	50	438–441	36
	Ccoc-R	CTTTGAGTTTCATTCTTGCGAA			
Faecalibacterium prausnitzii	Fae-F	GGAGGAAGAAGGTCTTCGG	60	248	38
	Fae-R	AATTCCGCCTACCTCTGCACT			
Roseburia spp.	Ros-F	GCGGTRCGGCAAGTCTGA	60	81	38
	Ros-R	CCTCCGACACTCTAGTMCGAC			
Prevotella	Pre-F	GAAGGTCCCCCACATTG	103	60	38
	Pre-R	CGCKACTTGGCTGGTTCAG			

### Statistical analysis

Enumeration data are tested by chi-square. Independent-samples T test was used to analyse the quantitative data. Spearman rank correlation were calculated to estimate the linear correlations between variables. Wilcox test, Kruskal-Wallis, PcoA, LEfSe, Monte Carlo test CCA were used to analyse the sequcing datas. Canonical correspondence analysis (CCA) was performed to measure physiological properties that have the most significant influence on microbial communities. Statistical analyses were performed with the statistical software package SPSS13.0 (SPSS Inc., Chicago, IL, USA). P < 0.05 was considered indicative of statistical significance.

## Results

### Patients and controls

CysC, BUN, and creatinine was significantly higher, and eGFR was reduced in ESRD patients compared to healthy controls. Levels of the plasma inflammatory biomarker CRP differed significantly between ESRD patients and controls (p = 0.005). LPS was increased in ESRD patients (p = 0.033). The ethic background of all the participants were Han nationally Chinese. All the ESRD patients had been treated with phosphate binders, oral iron supplements or intravenous iron compounds, antihypertensive drugs. 12 of the ESRD patients had been treated with calcium supplements and Vitamin D. There were no significant differences in age, sex, body mass index (BMI), glucose, TG, CHOL, VLDL, LDL, HDL, Lpa, Lipoprotein a (Lpa), apolipoprotein E (ApoE), and apolipoprotein A, B (ApoA, B) (Table 1).

### Diversity and phylum/subfamily -level taxonomic distribution of gut microbiota in ESRD patients

**Figure 1:**
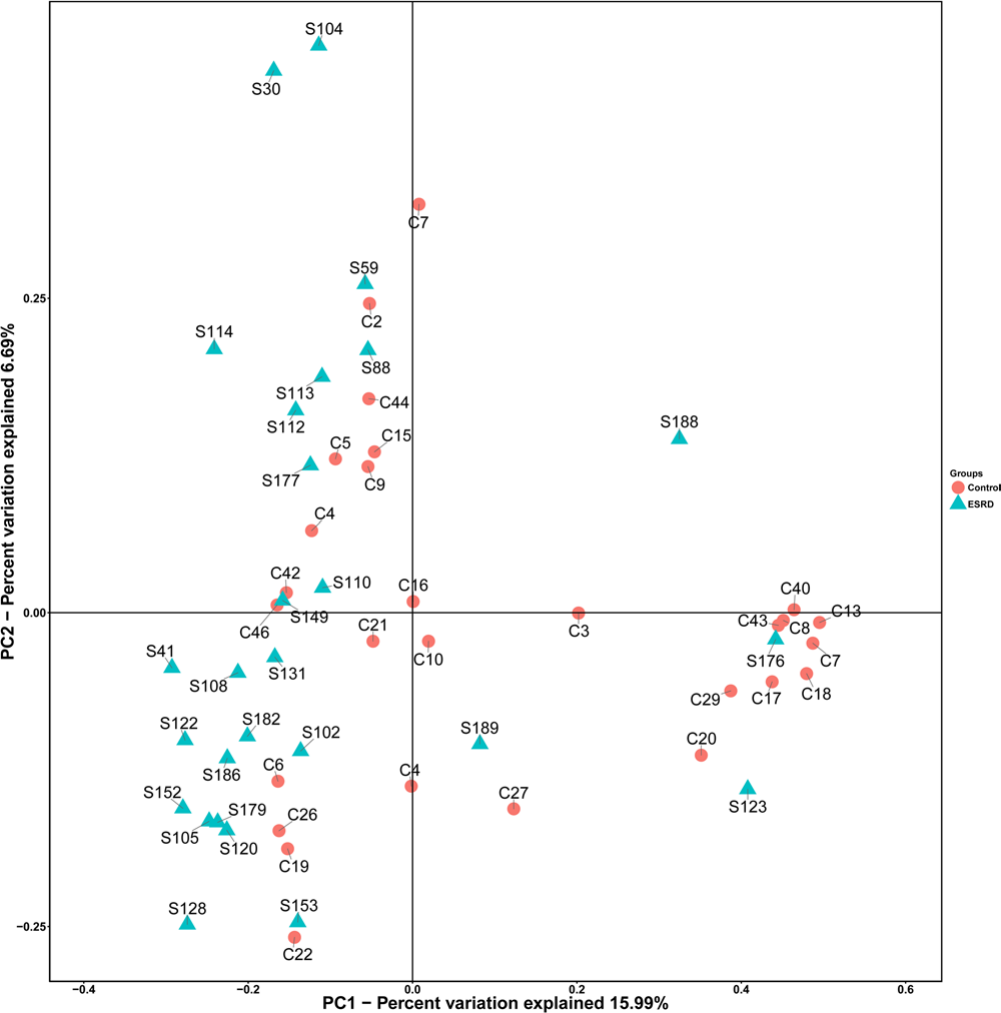
Principle Coordinate Analysis (PCoA) of gut microbiota from ESRD (Green) and healthy control (Red). The first two axes of the principal coordinate analysis are represented with principal coordinate axis 1 (15.99% variability) and principal coordinate axis 2 (6.69% variability).

Diversity concerns both taxon richness and evenness, and our results demonstrated that the diversity was similar (*p* > 0.05) as assessed by chao1, observed_species, Shannon, simpson diversity indexs. PCoA based on the UniFrac metric did not reveal a separation trend of healthy controls and ESRD patients (Fig. 1).

**Figure 2:**
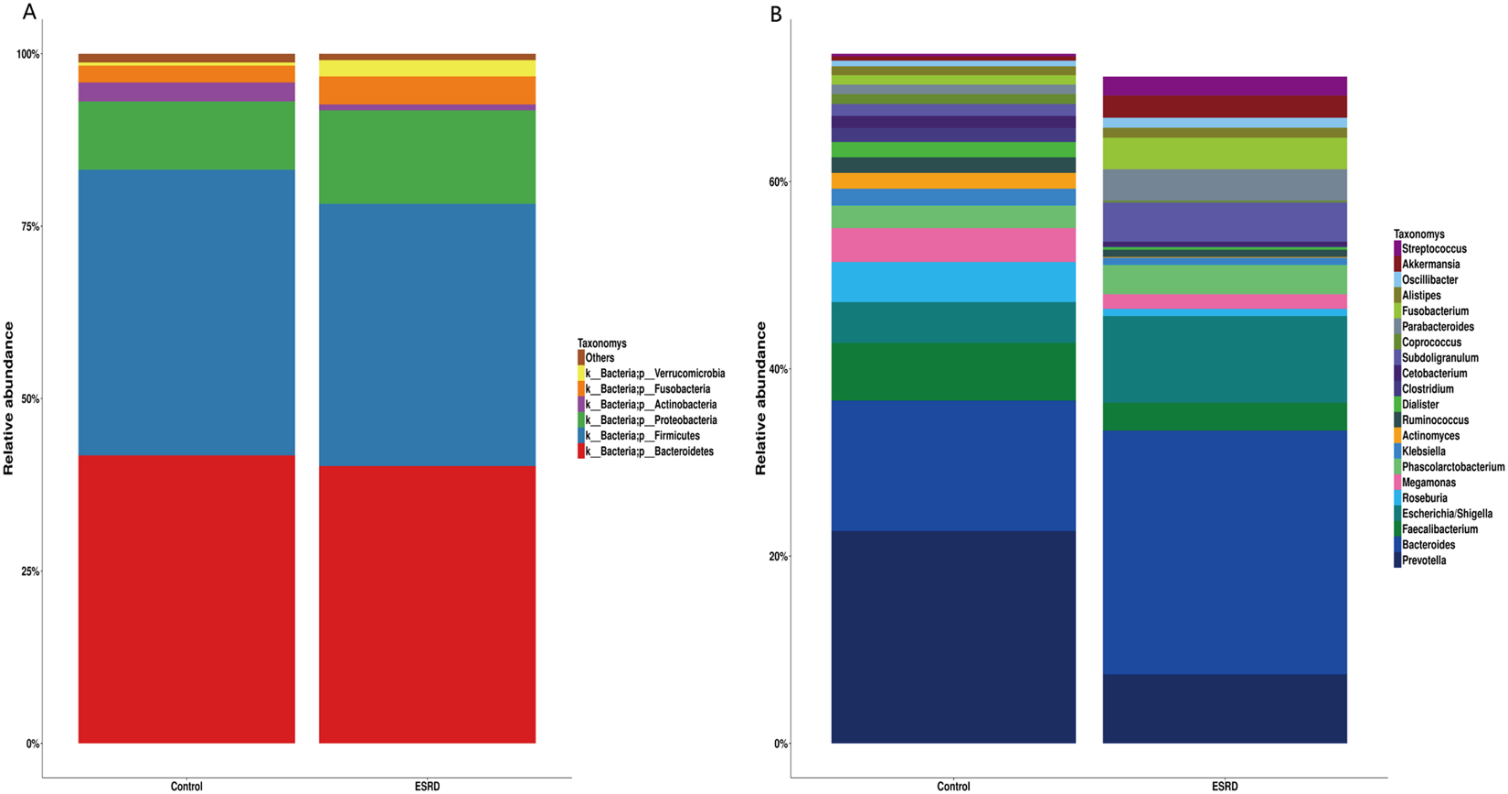
Relative abundance of the gut microbiota in this study. Microbiome composition in human from controls (n = 26) or patients with ESRD (n = 27). The composition is based on 16S rRNA sequecing. Results are shown at the (**A**) phylum and (**B**) genus level. The figure shows species median relative abundance >= 1% of total abundance in either the healthy control group or the ESRD group, and value < 1%, unclassified, unidentified are classified as Others.

Bacteroidetes was the most abundant phylum in both healthy individuals and CKD patients, accounting for 41.76%, 40.23% of the total valid reads respectively. Firmicutes was the second most abundant phylum in all samples with an average relative abundance of 41.43%, 38.01% respectively. The other dominant phyla were Proteobacteria, Actinobacteria, Fusobacteria, Verrucomicrobia and Others (Fig. 2A). Based on the average relative abundance, 21 genera were dominant (>=1%) at the genus level. Bacteroides, Escherichia/Shigella, Subdoligranulum, Fusobacterium. etc were enriched in ESRD patients. Prevotella, Roseburia, Faecalibacterium, Megamonas. etc were more abundant in controls (Fig. 2B).

### A reduction in SCFAs producing bacteria as a prominent feature of ESRD patients

**Figure 3:**
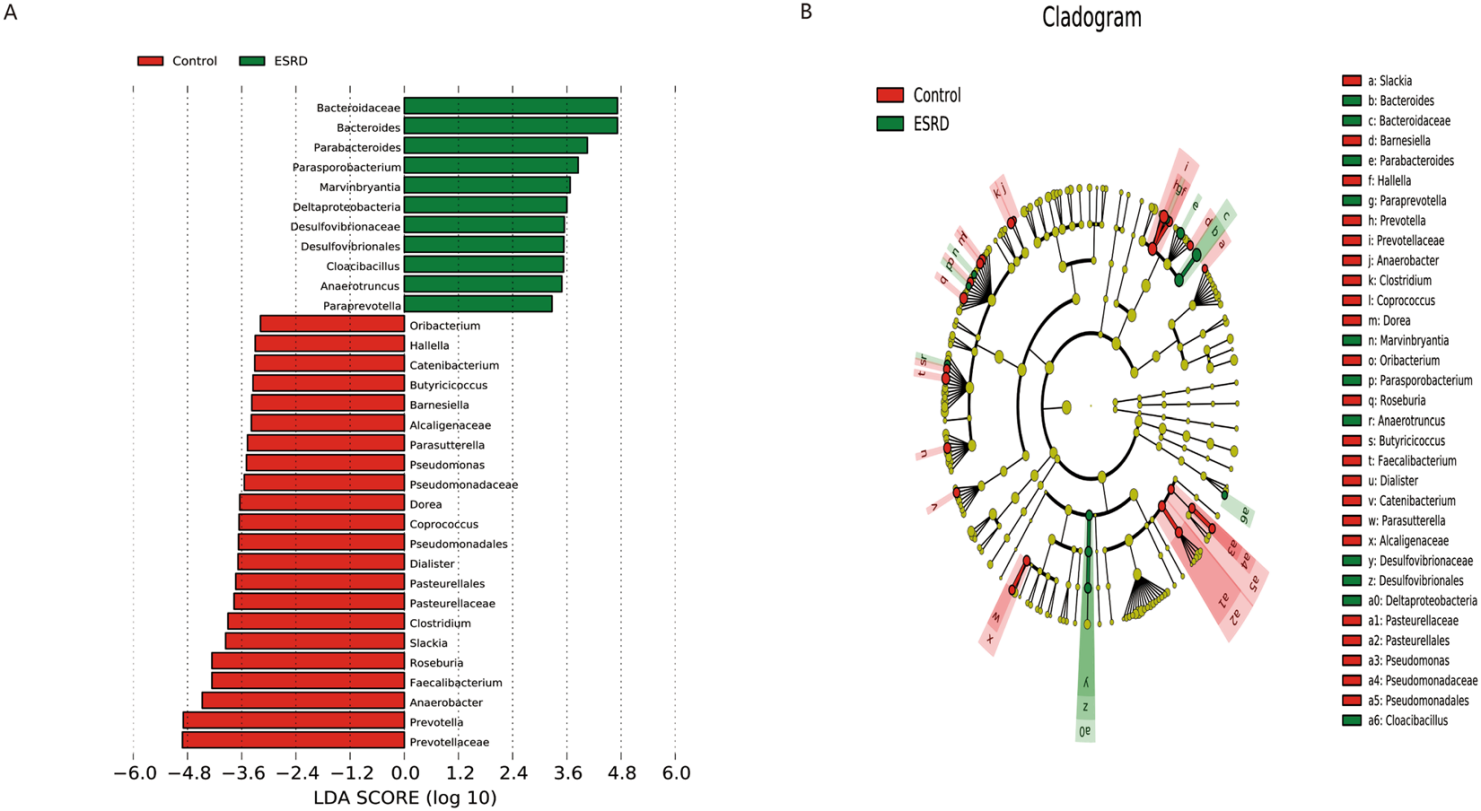
Indicator microbial groups within the three groups of individuals with logarithmic linear discriminant analysis (LDA) score higher than 2 determined by effect size (LefSe). (**A**) Histogram of the LDA scores computed for differentially abundant bacterial taxa between healthy controls and patients with ESRD. (**B**) Cladograms of bacterial lineages with significantly different representation in humans with or without ESRD. Lineages on the bacterial trees are color-coded to indicate whether the taxon does (red or green) or does not (yellow) significantly differ between sample classes. Of those, 11 bacterial taxa were significantly overrepresented in samples from ESRD (green) and 22 bacterial taxa were overrepresented in samples from healthy control (red). Prevotella (red) were significantly overrepresented in control and Bacteroides (green) were overrepresented in ESRD patients. The producing butyrate bacterial taxa (Roseburia, Faecalibacterium, Prevotellaceae, Prevotella, and Coprococcus) was under-abundant in ESRD patients.

LEfSe showed so much biomarkers for ESRD patients and controls subiects (Fig. 3) (LDA score > 2.0, p < 0.05). 11 species enriched in ESRD patients, and 22 in controls. According to Wong J^39^ Kyoto Encyclopedia of Genes and Genomes (KEGG) analysis, Bacteroidaceae with p-Cresol production enzymes enriched in ESRD patients. Desulfovibrionaceae, Bacteroidaceae, Alcaligenaceae, Pseudomonadaceae, and Pasteurellaceae produced urease, Bacteroidaceae’s relative abundance was higher, the others were much lower in ESRD patients than the controls group in this study. Microbes of the genus Prevotella, Roseburia, Faecalibacterium, Clostridium, Coprococcus can produce butyrate^26^, Dorea was the other predominant SCFA-producing genera^40^. All of these species were reduced in ESRD patients, indicating that bacteria producing SCFAs especially butyrate were decreased in ESRD patients. Bacteroides (enterotype 1), Prevotella (enterotype 2) and Ruminococcus (enterotype 3) were three main enterotypes of human gut microbiota^41^. In this study, from healthy people to ESRD patients, the enterotype changes from Prevotella (enterotype 2) to Bacteroides (enterotype 1).

### Canonical correspondence analysis (CCA)

**Figure 4:**
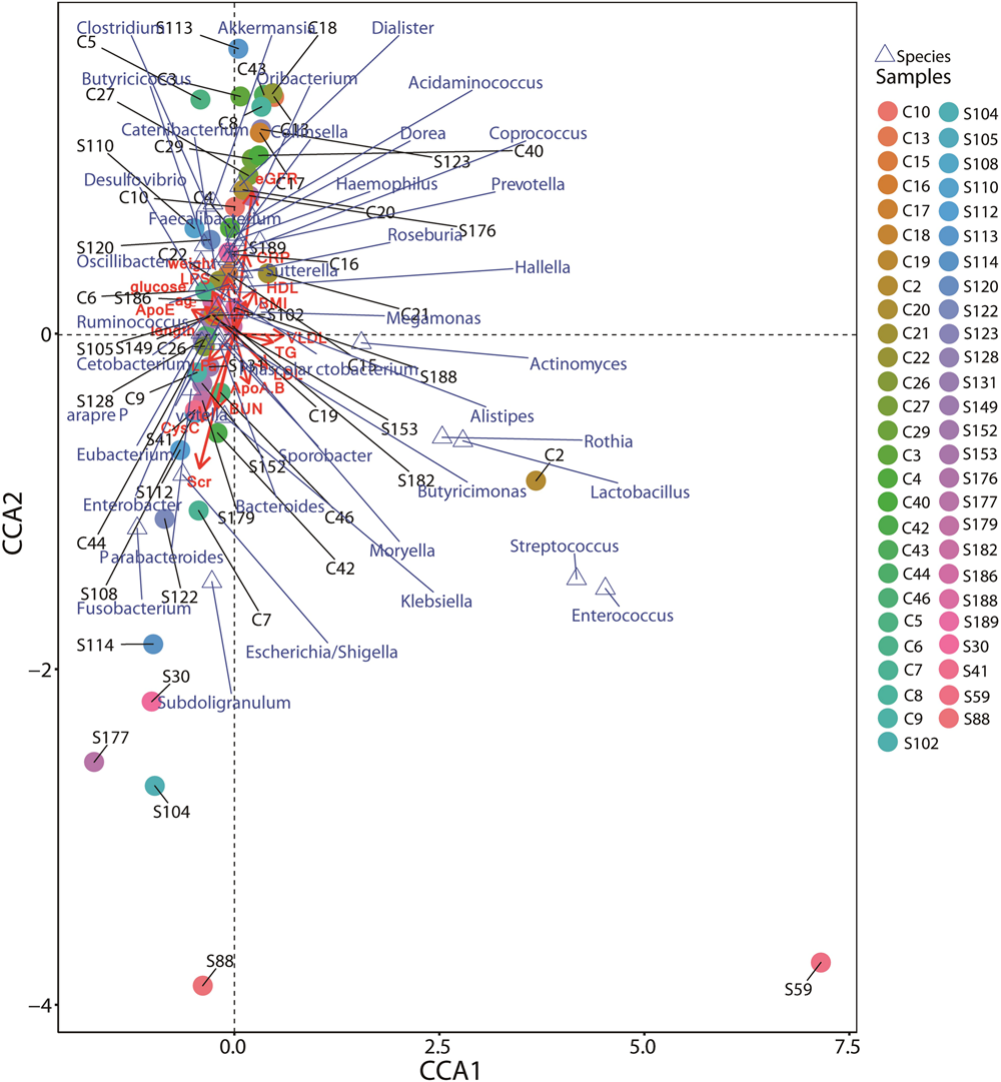
Canonical Correspondence Analysis (CCA) illustrating relations between bacteria taxa and internal environmental parameters accumulated in healthy controls and patients with ESRD in China. Arrows indicate the direction and magnitude of internal environmental parameters associated with bacterial community structure. The explained variance of the principal axes [Axis 1 (horizontally) and Axis 2 (vertically)] are 9.319% and 6.008%, respectively; The species explained 39.82% of the total information amount. The filled circle represents samples, triangle represents species. S* and C* represent ESRD patients and healthy controls respectively.

Microbial community may be more correlated with indigenous environmental parameters. Analyzing the dynamic changes of microbial communities with geochemical factors will reveal the correlation between environmental parameters and microbial community. Therefore, CCA analysis was used to reveal how microbes can adapt to the changes of physiochemical environments. A correlation between the important environmental parameters and microbial community was discerned by CCA analysis as shown in Fig. 4. Sixteen environmental parameters and the dominant genera (>1%) in each sample were selected to determine their correlation. The length of an environmental parameter arrow indicated the strength of the environmental parameter to the overall microbial communities. As such, CysC (r^2 = 0.1689, p = 0.020), creatinine (r^2 = 0.1593, p = 0.008) and eGFR (r^2 = 0.1255, p = 0.041) concentrations appears to be the most important environmental parameters (Monte Carlo test). For instance, Enterobacter, Bacteroides, Fusobacterium, Escherichia and Klebsiella, which were positively correlated with CysC, creatinine (Scr) as shown in Fig. 4, and dominant in ESRD patients. Whereas Faecalibacterium, Akkermansia, Prevotella, Roseburia, Coprococcus and Clostridium were positively correlated with eGFR, and dominant in controls. Therefore, it is fair to propose that CKD played an active role in shaping the indigenous microbial communities.

### Quantification of well known species in faeces by qPCR

**Figure 5:**
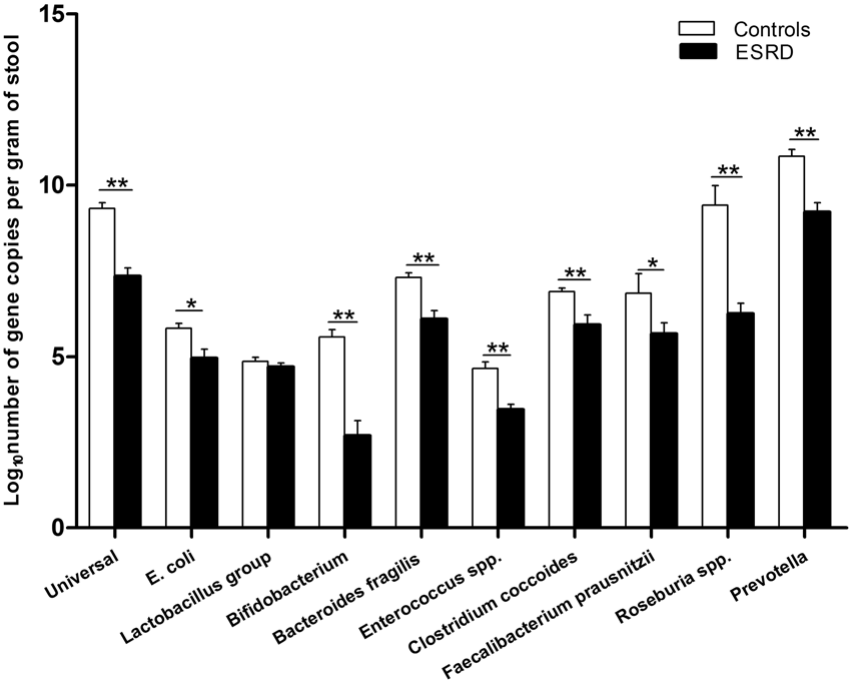
Bacterial groups quantified by qPCR expressed as log10 bacteria per gram of stool. qPCR for the common micropopulation in control subjects and ESRD patients. Black and white bars represent ESRD patients and healthy controls respectively. Independent-samples T Test was used to evaluate statistical difference between the two groups. *p < 0.05, **p < 0.01.

qPCR was used to assess changes in bacterial absolute quantity in faecal samples from the two groups (Fig. 5). Bacterial copy number values were converted into logarithmic values before analysis. Quantities of total gene copies of Universal bacteria, *E. coli*, Bifidobacterium, Bacteroides fragilis group, Enterococcus spp., Clostridium coccoides group, Faecalibacterium prausnitzii, Roseburia spp. and Prevotella were significantly decreased in ESRD patients compared with controls (p = 0.000, p = 0.001, p = 0.000, p = 0.000, p = 0.000, p = 0.000, p = 0.028, p = 0.000, p = 0.000, respectively). However, the numbers of beneficial microorganisms from the Lactobacillus group were similar between two groups (p = 0.395). In ESRD patients, universal bacteria were decreased, and the butyrate producing species Clostridium coccoides group, Faecalibacterium prausnitzii, Roseburia spp. and Prevotella were also reduced, consistent with the sequencing results.

### Butyrate producing gut microbes are negatively related to microinflammation and renal function via qPCR

Among these, the butyrate producing species Roseburia spp., Faecalibacterium prausnitzii, Prevotella and Universal bacteria, were negatively related to inflammation index CRP (r = −0.452, p = 0.001; r = −0.431, p = 0.002; r = −0.480, p = 0.000; and r = −0.438, p = 0.000; respectively) (Table 3). In addition, levels of Roseburia spp., Faecalibacterium prausnitzii, Clostridium coccoides group, Prevotella and Universal bacteria, were negatively correlated with CysC level sensitive index to evaluate glomerular filtration rate (r = −0.414, p = 0.003; r = −0.395, p = 0.005; r = −0.400, p = 0.001; r = −0.441, p = 0.001 and r = −0.493, p = 0.000; respectively); The Bifidobacterium and Universal bacteria were negatively associated with renal function index BUN and creatinine (r = −0.495, p = 0.000; r = −0.449, p = 0.000 or r = −0.538, p = 0.000; r = −0.519, p = 0.000, respectively). An inverse tendency was observed with regard to eGFR (r = 0.466, p = 0.000 and r = 0.511, p = 0.000). These findings strengthened and further proved the importance and necessity of butyrate producing bacteria in inflammation or renal function of CKD patients. These data further clarified that the structural dynamics of the bacterial community in the intestinal tract played an important role in CKD progression, especially the beneficial species.

**Table 3:** Correlation analysis of CRP, CysC, BUN, creatinine, eGFR values and the species count determined by qPCR.

		Roseburia spp.	Faecalibacterium prausnitzii	Clostridium coccoides group	Lactobacillus group	Bifidobacterium	Bacteroides fragillis	*E. coli*	Enterococcus	prevotella	Universal
CRP	r	−0.452**	−0.431**	−0.289**	−0.085	−0.303**	−0.277*	−0.295	−0.062	−0.480**	−0.438**
	p	0.001	0.002	0.008	0.447	0.005	0.015	0.075	0.538	0.000	0.000
CysC	r	−0.414**	−0.395**	−0.400**	−0.325*	−0.295*	−.0240	−0.116	0.040	−0.441**	−0.493**
	p	0.003	0.005	0.001	0.014	0.021	0.075	0.377	0.759	0.001	0.000
BUN	r	−0.237	0.083	−0.106	−0.018	−0.495**	−0.257**	−0.228*	−0.237*	−0.011	−0.538**
	p	0.082	0.545	0.268	0.851	0.000	0.008	0.024	0.012	0.935	0.000
creatinine	r	−0.070	0.049	−0.145	−0.064	−0.449**	−0.326**	−0.183	−0.264**	0.078	−0.519**
	p	0.609	0.724	0.172	0.524	0.000	0.001	0.097	0.008	0.559	0.000
eGFR	r	0.295*	0.117	0.243**	0.039	0.466**	0.260**	0.159	0.190*	0.287*	0.511**
	p	0.027	0.391	0.009	0.679	0.000	0.006	0.115	0.042	0.027	0.000
LPS	r	−0.392**	−0.298*	−0.179	−0.263*	−0.153	−0.068	−0.166	−0.035	−0.198	−0.127
	p	0.003	0.029	0.141	0.027	0.204	0.591	0.171	0.773	0.151	0.290
glucose	r	0.023	−0.247	−0.023	−0.274	−0.058	0.079	−0.056	0.131	−0.349	−0.226
	p	0.906	0.189	0.892	0.095	0.727	0.663	0.742	0.433	0.054	0.167
ApoE	r	−0.152	−0.300	−0.237	−0.252	−0.219	−0.327	−0.260	0.077	−0.074	−0.328
	p	0.474	0.054	0.208	0.172	0.236	0.083	0.159	0.692	0.729	0.071
ApoA, B	r	0.075	0.096	−0.022	0.219	0.258	0.079	0.028	0.296	−0.069	0.167
	p	0.728	0.661	0.909	0.119	0.67	0.684	0.883	0.119	0.749	0.370
Lpa	r	0.123	−0.243	−0.078	0.115	−0.038	0.125	0.149	0.066	−0.253	−0.162
	p	0.567	0.264	0.680	0.539	0.840	0.518	0.425	0.732	0.233	0.384
VLDL	r	−0.132	−0.145	0.039	0.155	−0.011	0.161	−0.076	0.263	−0.104	−0.042
	p	0.389	0.338	0.788	0.259	0.935	0.245	0.576	0.052	0.495	0.754
LDL	r	0.119	0.056	0.235	−0.014	0.049	0.137	0.062	−0.062	0.018	0.187
	p	0.476	0.734	0.096	0.922	0.773	0.337	0.817	0.653	0.918	0.165
HDL	r	0.083	0.114	0.040	−0.289	−0.020	−0.029	0.183	−0.242	0.200	0.173
	p	0.586	0.464	0.771	0.159	0.880	0.437	0.178	0.875	0.187	0.581
TG	r	−0.134	−0.043	0.088	0.268	0.031	0.067	−0.041	0.336*	0.009	0.003
	p	0.564	0.780	0.520	0.063	0.897	0.635	0.763	0.021	0.955	0.984
CHOL	r	0.078	0.006	0.202	−0.020	0.029	0.105	0.042	−0.042	0.073	0.239
	p	0.639	0.970	0.136	0.885	0.828	0.461	0.760	0.759	0.678	0.123

Abbrevitions: CysC, Cystatin C; BUN, Blood Urea Nitrogen; eGFR, estimated glomerular filtration rate. Spearman rank correlation were used to evaluate statistical importance: r: correlation coefficient. *p < 0.05, **p < 0.01.

## Discussion

This report represents the first investigation of faecal microbiota diversity and quantity among Chinese CKD patients that employ high-throughput sequencing and qPCR analyses. We supplemented the intestinal bacteria data of CKD patients. In the analysis of sequencing data, we did not find any diversity differences between CKD patients and controls, which suggests that the diversity of the bacterial community was not destroyed critically, it was not like microbe-scarce scenario. Bacteroidetes (~40%), Firmicutes (~40%) and Proteobacteria (~10%) were the predominant phyla in both healthy individuals and CKD patients, consistent with reports from previous studies among cohorts from Western countries, Africa and Asia^42–44^. Although Bacteroidetes and Firmicutes were the two most abundant phyla constituting the vast majority of gut microbiota in this study, an interesting variation occurred with regards to Bacteroidetes. Through LEfSe analysis, we found that Prevotella was enriched in the healthy group, and Bacteroides in the CKD group. This enterotype conversion proves once again the correlation between the intestinal flora and CKD^41^.

Distribution of a number of genera could be differentiated between ESRD patients and controls. The SCFAs (propionate, acetate, and butyrate) are a by-product of the fermentation of non-absorbable complex carbohydrates. Firmicutes- Clostridiales- Lachnospiraceae -Dorea producing SCFAs^40^ were diminished in ESRD patients. Members of Prevotellaceae possess phosphotransbutyrylase and butyrate kinase^39^, and Prevotella can produce SCFAs^45^. In this study, both Prevotella and Prevotellaceae were reduced in ESRD patients. The human colonic butyrate (Short-chain fatty acids) producers are Gram-positive firmicutes, but are phylogenetically diverse. *Clostridiales cluster* the XIVa (*Clostridium coccoides*) including Ruminococcus, Coprococcus, *Eubacterium hallii (E*. *hallii)*, *Eubacterium rectale/Roseburia spp*. and *Clostridiales cluster* IV (*Clostridium leptum*) including *Faecalibacterium prausnitzii*, and *Eubacterium* spp. are normally the two most abundant groups of human faecal bacteria that produce butyrate^26, 46, 47^. Roseburia, Coprococcus, and Faecalibacterium belong to Firmicutes-Clostridiales. All of them are typically producing butyrate bacteria and were particularly and significantly more abundant in healthy controls and decreased in ESRD patients and consistent with previous studies^39^. The qPCR analysis of Roseburia spp. and Faecalibacterium prausnitzii showed a similar trend in ESRD. Butyrate gets involved in the adjustment of body reaction to inflammation^29^. Systemic inflammation in patients with end-stage renal disease (ESRD) is mediated by activation of the innate immune system^48^. The presence of persistent inflammation magnifies the risk of poor outcome, and is a risk factor for cardiovascular disease (CVD), via mechanisms related to exacerbation of both wasting and vascular calcification processes and self-enhancement of the inflammatory cascade^49^. High dietary total fiber intake is associated with lower risk of inflammation and mortality in kidney disease^50^. Interestingly, CRP was increased in ESRD compared with that in controls. Spearman rank correlation analysis demonstrated that the absolute abundance of Roseburia spp., Faecalibacterium prausnitzii, Prevotella and Universal bacteria were negatively associated with CRP level and renal function indexes. These data indicate that bacteria producing butyrate as biomarkers may involve in the pathological process of CKD. Recently, Andrade-Oliveira V proved that SCFAs can reduce inflammation in acute kidney injury (AKI), which supports our inferences^51^.

Reduced quantity of fecal microbiota were found in ESRD patients on qPCR analysis, This means that the absolute quantity of total faecal microbiota was decreased in CKD patients. In general, Universal bacteria, *E. coli*, Bifidobacterium, Bacteroides fragilis group, Enterococcus spp., Clostridium coccoides group, Faecalibacterium prausnitzii, Roseburia spp. and Prevotella were decreased in ESRD. *Bifidobacterium*, *Roseburia* and *Clostridium coccoides* ^45^ can produce SCFAs. *Bacteroides fragilis* and *Clostridium* spp. can protect against dextran sulfate sodium (DSS)-or trinitrobenzenesulfonic acid-induced colitis^52, 53^. This suggests that CKD status may influence the absolute quantity of the microbiome, which may result from accumulation of uremic toxins, inflammation and malnutrition and needs further investigation. This reduction in beneficial bacteria may play an important role in the pathogenic processes of CKD.

LPS is derived from the cell wall of gram negative bacteria, and the increase of the gamma Proteobacteria is also effective in increasing the LPS level in circulation. The degree of circulating endotoxemia might be related to the severity of systemic inflammation and features of atherosclerosis in peritoneal dialysis (PD) patients^54^. LPS may accelerate activation of neutrophils and macrophages/monocytes, which further explain the persistent inflammation of ESRD^55^. Although most CKD patients presented signs of fluid overload that was associated with endotoxaemia, there was no association between endotoxaemia and systemic inflammation, suggesting the endotoxaemia may not be the main determinant of the inflammatory status in CKD patients^56^. So the correlation between LPS and inflammation is unclear. In this study, LPS was elevated in ESRD patients, but we didn’t find the correlation between LPS and bacterial amounts. To determine the characteristics of gut microbiota based on kidney function, we excluded the influences of body mass index (BMI), blood lipids, and blood glucose. No significant differences in blood lipid and blood glucose levels were found between CKD patients and controls consistent with that reported in previous study by McIntyre, C. W.^57^. Further research is needed in this area to provide more conclusive evidence while taking into account the relationships of gut flora with human diet, environment and habits.
